# Drug addictions and sexual violence in childhood and adolescence: analyzing life stories

**DOI:** 10.1186/1940-0640-8-S1-A45

**Published:** 2013-09-04

**Authors:** Andréa Marques Leão Doescher, Andreza Marques de Castro Leão, Paulo Rennes Marçal Ribeiro

**Affiliations:** 1 Department of Neuroscience, National Institute for Space Research, São José dos Campos, Brazil; 2 Faculty of Arts and Letters, University of São Paulo, Araraquara Campus, Araraquara, Brazil

## 

The sexual violence (SV) suffered in childhood and adolescence significantly affects the physical and mental health of the victims who are subjected to pain, shame and guilt, requiring them to adopt coping strategies of these feelings. The use of alcohol and drugs is a recognized way of dealing with the pain of living. This work of narrative feature analyzes and discusses through the life story of eight people, chemical dependency as a result of VS suffered in childhood or adolescence. The participants of this study are male and have suffered sexual violence in this age period of life. Their ages range from 23 to 39 years, and all are admitted to a therapeutic community in a city in the interior of Sao Paulo state, in Brazil, for treatment of chemical dependency, being met by the department of Psychology. Seven participants cited the violence suffered in childhood (between 7 and 9 years), and adolescence (age 14). The attackers were people closed to the victims. All patients claim that violence suffered influenced their entry into the world of drugs. Six participants declare themselves as homosexuals, a participant claims to have ceased to be gay and the other is not stated as a homosexual, but presents difficulties regarding sexuality. The participants see the drugs as an anesthetic of heartaches. Was noted that the patients have a double stigma in society: the issue of drugs addiction and the orientation of sexual desire, because the majority of participants are homosexual. The results reinforce the need for effective action geared to accommodate the victims of sexual violence and effective preventive measures to prevent them, as well as preventing the use of alcohol by adolescents victimized.

